# Comparative feasibility of implementing rapid diagnostic test and microscopy for parasitological diagnosis of malaria in Uganda

**DOI:** 10.1186/1475-2875-10-373

**Published:** 2011-12-19

**Authors:** Vincent Batwala, Pascal Magnussen, Fred Nuwaha

**Affiliations:** 1 Department of Community Health, Mbarara University of Science & Technology, P.O. Box 1410, Mbarara, Uganda; 2 Centre for Health Research and Development, Faculty of Life Sciences, Copenhagen University, Thorvaldsensvej 57, DK1871 Frederiksberg C, Denmark; 3 Disease Control and Environmental Health, Makerere University School of Public Health, P.O. Box 7072, Kampala, Uganda

## Abstract

**Background:**

In Uganda, parasite-based diagnosis is recommended for every patient suspected to have malaria before prescribing anti-malarials. However, the majority of patients are still treated presumptively especially in low-level health units. The feasibility of implementing parasite-based diagnosis for uncomplicated malaria in rural health centres (HCs) was investigated with a view to recommending measures for scaling up the policy.

**Methods:**

Thirty HCs were randomized to implement parasite-based diagnosis based on rapid diagnostic tests [RDTs] (n = 10), blood microscopy (n = 10) and presumptive diagnosis (control arm) (n = 10). Feasibility was assessed by comparing the proportion of patients who received parasite-based diagnosis; with a positive malaria parasite-based diagnosis who received artemether-lumefantrine (AL); with a negative malaria parasite-based diagnosis who received AL; and patient waiting time. Clinicaltrials.gov: NCT00565071.

**Results:**

102, 087 outpatients were enrolled. Patients were more likely to be tested in the RDT 44, 565 (96.6%) than in microscopy arm 19, 545 (60.9%) [RR: 1.59]. RDTs reduced patient waiting time compared to microscopy and were more convenient to health workers and patients. Majority 23, 804 (99.7%) in presumptive arm were prescribed AL. All (100%) of patients who tested positive for malaria in RDT and microscopy arms were prescribed anti-malarials. Parasitological-based diagnosis significantly reduced AL prescription in RDT arm [RR: 0.62] and microscopy arm [RR: 0.72] compared to presumptive treatment. Among patients not tested in the two intervention arms, 12, 044 (96.1%) in microscopy and 965 (61.6%) in RDT arm were treated with AL [RR: 1.56]. Overall 10, 558 (29.4%) with negative results [5, 110 (23.4%) in RDT and 5, 448 (39.0%) in microscopy arms] were prescribed AL.

**Conclusion:**

It was more feasible to implement parasite-based diagnosis for malaria using RDT than with microscopy. A high proportion of patients with negative malaria results are still prescribed anti-malarials. There is need to increase access to parasite-based diagnosis where microscopy is used. In order to fully harness the benefits of parasitological confirmation of malaria, it is necessary to reduce the prescription of anti-malarials in negative patients.

## Background

Malaria presents a diagnostic challenge in most endemic countries in sub-Saharan Africa, yet early diagnosis and appropriate treatment is a basic tenet of current malaria policy [1]. In sub-Saharan Africa, documentation of fever or history of fever has traditionally been considered sufficient evidence for prescribing anti-malarial therapy [2-4]. This presumptive diagnostic technique is inaccurate [5-8] and results in over diagnosis and over treatment. Further, because of the inaccuracies associated with presumptive treatment, decline in the proportion of fevers attributable to malaria, and use of expensive anti-malarials such as artemisinin-based combination therapy (ACT) [4,9-11], the World Health Organization (WHO) [1] now recommends parasite-based confirmation of parasitaemia before prescription of ACT.

The role of laboratory diagnosis of malaria is primarily to support clinical care [12], and the current reference standard for confirmatory presence of parasitaemia is microscopy. However, implementation of routine malaria microscopy is a challenge. Maintaining good quality, effective microscopy service requires an organized health system infrastructure, including the provision of high-quality supplies and reagents, presence of satisfactory microscopes, availability of microscopists, maintenance and technical competence of microscopists, an adequate workplace environment and the ability to prepare usable blood films. Field microscopy where established, often falls short of these requirements [13-15]. In Uganda, there is shortage of staff, especially laboratory personnel to perform microscopy [16].

Because parasite detection is performed by someone other than the prescriber, there is a tendency to ignore microscopy results provided by the laboratory [2-4]. Yet health workers' adherence to diagnostic and treatment guidelines is a critical aspect in determining effective implementation of malaria case management policies [17,18]. To put testing and clinical decisions in the hands of the prescriber, the use of malaria rapid diagnostic tests (RDTs) has been encouraged [1,7,10,15] as an integral part of widespread deployment of ACT. In line with international recommendations, Uganda introduced artemether-lumefantrine (AL) as first-line treatment for uncomplicated malaria in 2006, and commenced a phased role-out of a histidine rich protein-2 (HRP-2)-based RDT as an alternative to microscopy in primary level health centres (HCs) in 2008. However, quality parasite-based diagnosis remains unavailable to most outpatients presenting with febrile illness.

Although the sensitivity and specificity of malaria microscopy and RDTs have been assessed in different studies [8,19] and the feasibility of RDT in other settings [20], there has been no study gauging the feasibility of the use of these diagnostic strategies and whether the use of parasite-based diagnosis changes prescription of AL in rural settings in Uganda. The key question is "given the current standing at sub-county HCs and available resources, is it possible to test every patient presenting with febrile illness before treatment? Therefore the current study compared the feasibility of these malaria diagnostic techniques in rural government HCs located within areas of varying transmission intensities in Uganda. The main outcome measures were: proportion of febrile outpatients receiving a malaria test, proportion of those with negative results treated with AL and waiting time.

## Methods

### Study design

This stratified cluster randomized trial (Clinicaltrials.gov: NCT00565071) was implemented in 15 out of 20 sub-county government HCs randomly selected in a district of low and 15 in a district of high malaria transmission intensity from March 2010 to July 2011. Stratification was based on transmission intensity. HCs were the primary sampling units. In each district, HCs were randomized to two intervention arms (microscopy and RDT) and control arm (presumptive diagnosis), resulting into five HCs per arm. This trial took a cluster design to avoid contamination between control and intervention group subjects and to allow service providers to operate as they would normally on a day-to-day basis.

### Setting

The trial was carried out in Bushenyi and Iganga districts in Uganda, and commenced before the two districts were partitioned. However, partitioning did not affect the delivery of health services by the time the study was closed. Bushenyi district has a population of 731, 392. The district experiences low and unstable malaria transmission, with people of all ages being at risk. It is epidemic-prone, with occasional malaria outbreaks occurring shortly after the rains. Iganga district has a total population of 540, 939 with 15 HCs at sub-county level. The district experiences very high malaria transmission intensity. Malaria is the leading cause of morbidity and outpatient attendance for all age groups. Additional description of the study setting has been published elsewhere [16].

### Study procedures

#### Randomization of health centres to the three arms

Health centres were allocated to the three diagnostic arms following simple randomization. In Bushenyi district, three of the five HCs randomized to the microscopy arm were already offering the service. The study strengthened the laboratory of Kabira HC with a microscope and supplies. In Iganga district, two HCs were offering microscopy services but the study strengthened the laboratory for one (Nambale HC). In the other three HCs, the posts for laboratory personnel were already filled although the laboratories were not functional. Therefore, the district provided microscopes and laboratory supplies. In the RDT arm, three selected HCs in Bushenyi district and two in Iganga had microscopy services. However, RDTs were introduced and both clinicians and laboratory personnel were trained. They were informed during training that their HCs had been randomized to use RDTs. However, they were free to test patients with microscopy if needed. In the presumptive arm although one HC in Bushenyi and two in Iganga had the laboratory assistants post filled, the laboratories were not functional. They either had no space or laboratory equipment and supplies.

#### Delivery of RDTs and artemether-lumefantrine (AL)

Before the study commenced RDTs, AL, laboratory reagents and supplies from the National Medical Stores were delivered to the districts medical stores. This was meant to integrate the study into the district health services delivery system. It was also a strategy for continuity of services from the time of closing the study. RDTs were packaged in cartons of 1, 000 tests. In each carton, there were 40 boxes of 25 tests. Also in the box were 25 alcohol swabs, lancets and a 10 ml bottle of clearing buffer. Each sachet of RDT contained the test device, a loop for collecting blood and a descant. The study HCs were to make requisitions for AL, RDTs, and laboratory supplies together with antibiotics, analgesics and sundries following the usual guidelines. HCs opened up stock cards for RDTs that were updated regularly.

#### Training of staff

Clinicians (clinical officers, nurses/midwives and nursing assistants) and laboratory assistants received a one-day refresher training on-site. The training and subsequent study procedures were a scale-up of the activities performed during the assessment of the accuracy of these diagnostic techniques [8]. All staff members were trained in theory by re-orienting them to the malaria treatment policy. In RDT arm, the staff were in addition trained in 1) finger prick for collection of blood and 2) preparation and reading of *Paracheck^®^*. In the microscopy arm, members were in addition trained in: 1) finger prick for collection of blood, 2) thick/thin blood smear preparation, 3) staining smears, and 4) blood smear reading. In the microscopy and RDT arms, data collection commenced after inter-reader reliability reached a very good agreement (kappa coefficient = 0.97). The staff in HCs with microscopy or RDT were instructed to treat patients who have positive results with anti-malarials. Those with negative results were to receive alternative medications after further assessment. Staff in the control arm were only re-oriented to the current malaria treatment guidelines. HC outpatient registers were modified to record additional variables such as the presenting complaints, drugs dispensed and to indicate those prescribed but out-of-stock. The trained staff were charged with training those that were off-duty on the day of training. Further clarifications were provided during supervision. Supervision by the study team and the district laboratory focal persons was carried out weekly during the first two months and monthly thereafter. The supervisory visits were aimed at troubleshooting problems related to the skills of performing an RDT and interpretation of results, quality of the test, quality of blood smears, reagents, status of the microscopes and recording of results. In addition to monthly support supervision, the district laboratory focal person provided further visits during the routine quarterly schedules.

### Description of the diagnostic arms

#### Presumptive (control) arm

Patients presenting with fever (by statement or measured) were enrolled to receive service without parasitological confirmation of malaria. Patients were treated on the basis of signs and symptoms only.

#### Microscopy arm

Patients were enrolled on the basis of fever (by statement or measured). Microscopy was performed by laboratory assistants. These laboratory personnel have two to three years of pre-service training. Thick and thin blood smears were prepared by finger-prick using sterile blood lancets on separate frosted slides. Standard staining was performed using the Field's stain method. Laboratory assistants were only familiar with this staining technique. Blood films were read at magnification x1, 000. Each film was graded as positive (asexual malaria parasites seen) or negative (no malaria parasites seen) based on inspection of 200 fields. Microscopy test results were recorded in the laboratory registers. Patients received the results and treatment on the same day of visit.

#### Rapid diagnostic test arm

Patients presenting with fever (by statement or measured) underwent rapid testing with the "*Paracheck^®^*" device (Orchid Biomedical Systems, Goa, India). *Paracheck Pf^® ^*is based on the detection of histidine rich protein-2 (Pf HRP-2) produced by *Plasmodium falciparum *trophozoites and young gametocytes. The specimens were drawn by trained clinicians or laboratory assistants using a simple finger-prick. The test preparation and interpretation were done following manufacturer's instructions and standard operating procedures prepared for this study. The test was considered positive when the antigen line was visible in the test window, negative when only the control band was visible. RDT results were recorded in the outpatient registers. Also patients received the results and treatment on that day of visit.

#### Duration of outpatient visit

The duration of outpatient visit was assessed in six HCs using a random sample of 1627 consenting patients. When a patient arrived at the HC, time was recorded by the research assistant on a "time sheet." The "time sheet" was then given to the patient. Thereafter, the time was recorded by clinicians and laboratory personnel at every point of service delivery. The "time sheet" was finally retained at the dispensing window. For the laboratory personnel, the effective contact time was comprised of: drawing a sample from the patient, slide preparation, scanning the 200 film fields until declaring a slide negative and reporting of results. With regard to RDT, the effective contact time was comprised of: drawing blood samples from patients, applying samples onto the test, test reading and reporting of results.

#### Overall data collection

Data collection was carried out weekly by the research assistants by extracting information from the laboratory and outpatient registers from March 2010 to July 2011.

#### Statistical analysis

The collected data were manually checked and cleaned. Data were double entered by two trained database assistants in a customized entry template with in-built consistency checks in EpiData 3.1 software (The EpiData Association, Odense, Denmark). The two data sets were validated to check for entry errors. Before analysis in Stata version 10 (Stata Corp LP, College Station, Texas, USA), the data was declared a cluster design using the "svyset command" with HCs as primary sampling units. Further, the Poisson regression model was fitted while accounting for clustering. Probability values (*p*-values) were set at 0.05 and confidence intervals (CI) were calculated at the 95% level. Socio-demographic and symptom data was presented using descriptive statistics: distribution by age, number and percent of patients with positive and negative results by transmission setting and diagnostic method and prescribed drugs.

#### Ethical considerations

The study was approved by Makerere University School of Public Health Higher Degrees Research and Ethics Committee; and the Uganda National Council for Science and Technology (Ref: HS 209). The study was registered with the Clinicaltrials.gov (NCT00565071).

## Results

### Description of the sample

**Figure 1:**
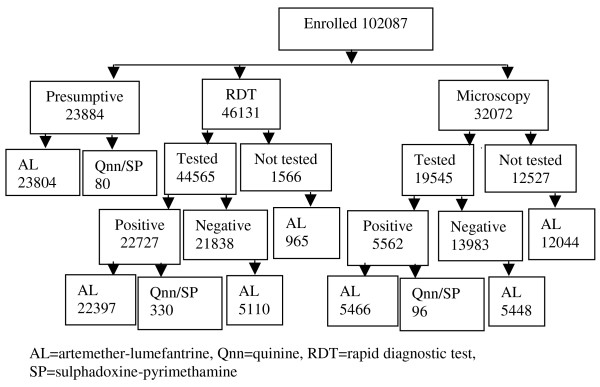
**Study profile**.

The study was carried out in 30 sub-county level government HCs. Each diagnostic arm had five HCs located in an area of low malaria transmission and another five in a setting of high transmission intensity. Overall, 102, 087 outpatients presenting with fever were enrolled. This included 52, 116 (51%) in low and 49, 971 (49%) in high transmission settings. Those enrolled in the presumptive arm were 23, 884, RDT 46, 131 and microscopy arm 32, 072 (Figure 1). Of the patients enrolled 59, 876 (58.7%) were females and 26, 421 (25.9%) were children under five years of age (Table 1). The median age in years for children under-five was 1.7 [inter-quartile range one to three years] and for those five years and above was 22 [inter-quartile range 13-35 years].

**Table 1:** Selected characteristics of study sample

Characteristic	Presumptive (%) [Int. range]	RDT (%) [Int. range]	Microscopy (%) [Int. range]
Level at the health delivery system	Sub-county	Sub-county	Sub-county
Number of health centres per arm	10	10	10
Total enrolment	23884	46131	32072
Gender (female)	14239(59.6)	26613(57.8)	19024(59.3)
Children under-five years of age	5265(22.0)	12828(27.8)	8328(26.0)
Median age (in years)			
< 5 years	2[1-3]	1.6[1-3]	1.7[1-3]
≥5 years	21[13-34]	23[14-35]	20[12-35]
Proportion with history of fever			
< 5 years	2695(94.0)	3393(87.5)	4224(88.0)
≥5 years	5568(73.7)	11936(70.3)	9276(68.4)

Int. range = inter-quartile range, RDT = rapid diagnostic test

### Proficiency in conducting the test

One hundred and thirty three HC staff were trained for the study. The staff included: 24 clinical officers, 13 nursing officers, 15 enrolled/registered nurses, 30 midwives, 36 nursing assistants and 15 laboratory assistants. Clinical officers have three years of pre-service training; nurses/midwives have two, laboratory assistants two to three years while nursing assistants have six to nine months. Laboratory assistants had two to six years of work experience. All cadres of staff had attended training in human immunodeficiency virus (HIV) counselling and testing. Therefore, they were experienced in testing HIV using whole blood on rapid test strips, devices or cassettes that work on the same principle as malaria RDTs. At this level of the health care services delivery system the staff in the RDT arm mentioned that one day of training is adequate, mainly focusing on practical (preparation of test and interpretation of results). On the first day, few staff especially nursing assistants had a challenge in collecting blood for the RDT using a loop in children under five years. However, they had gained adequate skills by the end of the first week. In the microscopy arm, all staff attended the practical session. Although they were able to prepare a thick blood smear, reading and interpretation of results was the responsibility of the laboratory personnel. Eight of the HCs (four in each district) in the microscopy arm did not have electricity. They used natural light when scanning for malaria parasites. In the remaining two HCs, black-out was frequent and electricity as source of light for malaria microscopy was unreliable.

### Duration of outpatient visit

The effective average contact time of clinicians with febrile outpatients (excluding time for investigation) was 11.4 min [95%CI: 11.1-11.7], and was similar in the three arms (Table 2). The mean effective contact time for malaria investigations was significantly shorter when using RDT 7.6 min [95%CI: 7.1-8.0] compared to microscopy 11.0 min [95%CI: 10.6-11.4]. On average, outpatients spent about two and half hours to complete a HC visit. However, the time spent accessing services in the RDT arm 134.4 min [95%CI: 123.5-145.3] was not different from that of presumptive treatment, but significantly shorter than when using microscopy 188.5 min [95%CI: 173.8-203.3].

**Table 2:** Mean patient time (in minutes) at different points of seeking care at government health centres

Variable	Presumptive Mean[95%CI]	RDT Mean[95%CI]	Microscopy Mean[95%CI]	Overall Mean[95%CI]
Contact with clinician	11.1[10.6-11.6]	11.9[11.3-12.5]	11.3[10.8-11.9]	11.4[11.1-11.7]
Contact for investigation	N/A	7.6[7.1-8.0]	11.0[10.6-11.4]	9.3[8.9-9.6]
Waiting time for test results	N/A	37.5[32.8-42.3]	123.9[105.9-142.0]	62.4[54.6-70.2]
Overall waiting time	135.6[123.0-148.2]	109.2[98.3-120.1]	156.1[141.4-170.9]	133.7[126.0-141.3]
Time spent at HC	143.8[131.2-156.4]	134.4[123.5-145.3]	188.5[173.8-203.3]	155.6[147.8-163.4]

CI = Confidence Interval, HC = health centre, N/A = not applicable, RDT = rapid diagnostic test

### Malaria test results

Overall, 64, 110 in the two intervention arms were tested (Table 3). The proportions tested in the two transmission settings were not statistically different [RR: 1.10; 95%CI: 0.87-1.40]. However, patients were 1.59 times more likely to have a malaria test done in the RDT arm 44, 565 (96.6%) [95%CI: 93.8-99.4] compared to the microscopy arm 19, 545 (60.9%) [95%CI: 47.9-74.0], [RR: 1.59; 95%CI: 1.31-1.92]. Still patients were more likely to be tested in the RDT arm compared to microscopy arm in children under-five years of age [RR: 1.56; 95%CI: 1.20-2.02], patients five years and above [RR: 1.59; 95%CI: 1.34-1.90], patients in low transmission area [RR: 1.45; 95%CI: 1.41-1.49] and in the high transmission setting [RR: 1.71; 95%CI: 1.06-2.76].

**Table 3:** Proportion of patients tested and malaria results by age and transmission intensity

Diagnostic method	Low transmission			High transmission		
	
	Enrolled	Tested n(%)	Positive n(%)	Enrolled n(%)	Tested n(%)	Positive n(%)
Presumptive						
< 5 years	2936	-		2329	-	
≥5 years	14035	-		4584	-	
RDT						
< 5 years	2716	2571(94.7)	583(22.7)	10112	10075(99.6)	7932(79.2)
≥5 years	14921	13624(91.3)	2730(20.1)	18382	18295(99.5)	11482(62.8)
Microscopy						
< 5 years	2900	1768(61.0)	269(15.2)	5428	3502(64.5)	2122(60.6)
≥5 years	14608	9313(63.8)	1279(13.7)	9136	4962(54.3)	1892(38.1)

RDT = rapid diagnostic test

Overall, 28, 289 (44.2%) [95%CI: 30.8-57.5] had a positive test result. In the low transmission setting, the proportion of those who tested positive in the RDT arm was 3, 313 (20.5%) [95%CI: 17.4-23.6] and microscopy arm 1, 548 (14.0%) [95%CI: 11.2-16.7]. The proportion of those testing positive in the RDT arm 19, 414 (68.6%) [95%CI: 62.2-74.9] in the high transmission setting was also significantly higher than in the microscopy arm 4, 014 (47.4%) [95%CI: 35.7-59.1]. Generally, the risk of having a positive test was higher among children under-five years of age 10, 900 (61.1%) compared to patients five years old and above 17, 124 (37.4%) [RR: 1.62; 95%CI: 1.41-1.86]. This relationship was maintained in the low transmission area [RR: 1.12, 95%CI: 1.05-1.21], and increased in the high transmission setting [RR: 1.29; 1.14-1.47].

### Treatment of patients who did not receive a parasitological diagnosis for malaria

All patients in the control arm were treated with anti-malarials (Figure 1 and Table 4). Prescription of AL in the control arm was not significantly different between those under five years and the older age group. A total of 12, 527 (39.1%) in microscopy and 1, 566 (3.4%) in RDT arms were not tested [RR: 11.50; 95%CI: 5.16-25.65]. Subsequently, 12, 044 (96.1%) and 965 (61.6%) respectively were treated with AL.

**Table 4:** Patients treated with artemether-lumefantrine stratified by diagnostic method, age and transmission intensity

	Low transmission				High transmission			
	**Presumptive** **n(%)** **[95%CI]**	**Microscopy** **n(%)** **[95%CI]**	**RDT** **n(%)** **[95%CI]**	**Total** **n(%)** **[95%CI]**	**Presumptive** **n(%)** **[95%CI]**	**Microscopy** **n(%)** **[95%CI]**	**RDT** **n(%)** **[95%CI]**	**Total** **n(%)** **[95%CI]**

**Not tested**								
Age (yrs)								
< 5	2932(99.9) [99.7-100]	1103(97.4) [95.6-99.2]	129(88.8) [80.1-97.9]	4164(98.8) [97.6-100]	2309(99.1) [97.9-100]	1842(95.6) [94.3-97.0]	33(89.2) [78.6-99.7]	4184(97.5) [95.6-99.3]
≥5	13999(99.7) [99.6-99.8]	5163(97.5) [96.5-98.4]	741(57.1) [54.9-59.3]	19903(96.5) [93.1-99.9]	4564(99.6) [99.4-99.7]	3936(94.3) [92.7-95.9]	62(71.3) [61.5-80.9]	8562(96.8) [94.1-99.5]
Total	16931(99.7) [99.6-99.8]	6266(97.5) [96.5-98.5]	870(60.3) [57.9-62.8]	24067(96.9) [93.9-99.8]	6873(99.4) [99.0-99.8]	5778(94.7) [93.5-95.9]	95(76.1) [69.0-84.2]	12746(97.0) [94.6-99.4]
**Tested**								
**Positive**								
Age (yrs)								
< 5	N/A	264(98.1) [95.2-100]	576(98.8) [98.4-99.2]	840(98.6) [97.8-99.4]	N/A	2106(99.3) [97.8-100]	7853(99.0) [97.6-100.]	9959(99.1) [98.1-100]
≥5	N/A	1254(98.0) [96.1-99.7]	2675(98.0) [97.4-98.6]	3929(98.0) [97.5-98.5]	N/A	1842(97.4) [97.8-98.8]	11293(98.4) [96.5-100]	13135(98.2) [96.9-99.5]
Total	N/A	1518(98.1) [96.9-99.2]	3251(98.1) [97.6-98.7]	4769(98.1) [97.7-98.5]	N/A	3948(98.4) [96.9-99.7]	19146(98.6) [96.9-100]	23094(98.6) [97.4-99.7]
**Negative**								
Age (yrs)								
< 5	N/A	374(25.0) [21.6-28.3]	332(16.7) [11.2-22.2]	706(20.3) [16.1-24.4]	N/A	1138(82.5) [80.4-84.5]	1246(59.6) [57.5-61.8]	2384(68.7) [44.0-93.4]
≥5	N/A	1630(20.3) [16.4-24.2]	1413(13.0) [12.4-13.6]	3043(16.1) [11.7-20.5]	N/A	2298(74.9) [73.3-76.4]	2055(30.2) [29.1-31.3]	4353(44.1) [40.2-48.9]
Total	N/A	2004(21.0) [17.4-24.7]	1745(13.6) [12.9-14.2]	3749(16.8) [12.4-21.1]	N/A	3436(77.2) [75.9-78.4]	3301(37.1) [36.1-38.1]	6737(50.5) [43.1-55.4]

CI = confidence interval, N/A = not applicable, RDT = rapid diagnostic test

### Treatment of patients with a positive RDT or microscopy

All patients in both microscopy and RDT arms with positive results received anti-malarial treatment (Figure 1). Overall, parasitological confirmation of malaria was associated with a reduction in the prescription of AL between presumptive and RDT [RR: 0.62; 95%CI: 0.47-0.82], and between presumptive and microscopy [RR: 0.72; 95%CI: 0.60-0.86].

### Treatment of patients with negative RDT or microscopy results

Overall, 10, 558 (29.5%) were treated with AL. Patients with RDT negative results were more often prescribed AL in the high transmission setting 3, 301 (37.1%) compared to those in the low transmission area 1, 744 (13.6%) [RR: 2.74; 95%CI: 1.05-7.15]. In the microscopy arm, there was a two to five-fold likelihood of prescribing AL to patients with negative results in the high transmission area 3, 436 (77.2%) than in the low transmission setting 2, 002 (21.0%) [RR: 3.67; 95%CI: 2.43-5.54]. This implies that the likelihood of not accepting negative microscopy results was higher than that for RDT in a setting of high malaria transmission.

Within the low transmission setting, children under-five years of age with negative microscopy results were more often prescribed AL compared to those in the RDT arm [RR: 1.49; 95%CI: 1.14-1.97]. A similar pattern in AL prescription was observed among patients five years and above. In the high transmission setting there was no statistically significant difference in AL prescription among children under-five years when those treated under microscopy were compared to those under RDT. Among patients five years and above in the high transmission setting 2, 055 (30.2%) in the RDT arm and 2, 298 (74.9%) in microscopy arm were prescribed AL [RR: 2.48; 95%CI: 0.83-7.41].

## Discussion

This study provides data on the comparative feasibility of microscopy and RDT among outpatients attending government rural primary health care centres located within areas of varying transmission intensities. The study findings indicate that RDT was more feasible than microscopy and prescribers were unlikely to adhere to negative results especially in the microscopy arm.

It is reported here that patients attending HCs with RDT as the method for malaria investigation had a higher probability of getting a parasitological test done regardless of age and transmission setting. Out of 133 clinical and laboratory staffs trained in 30 HCs, 56 were in the RDT arm and they performed the testing of patients during the whole period of study implementation. In the microscopy arm, the staff either had clinical or laboratory roles, although clinicians were able to prepare usable thick smears. Microscopy was the sole responsibility of the laboratory assistants. This meant that the RDT diagnostic services were not interrupted even if the laboratory assistants were away on leave. Performing RDT takes a much shorter time than microscopy. Therefore given a similar time, more patients could be tested in RDT compared to microscopy. RDTs reduced the patient waiting time compared to microscopy and were thus more convenient for health workers and patients. An earlier investigation [21] also reported a high number of patients tested by RDT. However, that study did not have a microscopy arm and therefore constraining a full comparison with the current findings. In Tanzania, a study that introduced routine use of malaria RDTs only resulted into 35% of patients being tested [22].

The risk of a febrile patient not getting a malaria diagnostic service was higher in HCs with microscopy and even far higher among patients under-five years of age. The current malaria treatment policy in Uganda and WHO [1] recommend that malaria case diagnosis be based on parasitological confirmation either by microscopy or RDT. An earlier publication reported a shortage of staff where only 34% of laboratory assistant posts were filled, although only four HCs had functioning laboratories at the time [16]. In order to improve the availability of microscopy services, there is need to functionalize the laboratories and to train and post at least two laboratory assistants at each HC. The low rate of malaria tests done in the microscopy arm adds to previous reports [13-15,23] regarding the microscopy limitations, signifying the difficulties surrounding its feasibility and scale up of the service.

With routine use of parasitological confirmation of malaria, prescription of AL was reduced by 28.1% between presumptive and microscopy; and by 38% between presumptive and RDT. This benefit was also reported in other studies [21,24], but it was offset by continued prescription of AL to patients with negative results. Indeed treatment of this "negative syndrome" with AL is a cause for concern in both intervention arms, and it was significantly higher in the microscopy arm in both transmission settings and age groups. This might imply prescribers were unlikely to adhere to negative microscopy results. Prescribing anti-malarials among patients with negative results has also cited [2-4,9,21,22,25-27]. The behaviour of treating negative patients with AL may reflect the hangover of previous practices of presumptive treatment, doubting accuracy of test methods, patients having been on anti-malarials before, or clinicians not knowing how to treat patients with negative results due to lack of clear guidelines. The continuous prescription of anti-malarials by clinicians disregarding negative test results is likely to impact on the cost-effectiveness of the diagnostic methods, clinical care of patients as well as increasing the costs of diagnosis and that of the overall treatment. Furthermore if negative patients continue receiving anti-malarials, health workers are more likely not to see the need for parasite-based diagnosis and may not be motivated to implement the policy. Therefore, service providers need support and guidelines on how to manage patients with negative results.

In the preparation for the study, RDTs, AL and laboratory supplies were delivered by the study team to the district medical stores. However, AL stock-out occurred in the high transmission setting that resulted into a reduction in the number of outpatient attendance. Subsequently this impacted on the number of patients enrolled in the high transmission setting. RDTs were in-stock throughout the study implementation period. The training of staff for this study took one day. However, other cadres of staff without such skills as HIV testing might require slightly longer period (three to five days). To acquire adequate skills for example in testing malaria with RDT, it is important to make frequent supervisory visits and tapering the number of visits with time. In this study, it was planned to have three diagnostic arms (presumptive, RDT and microscopy). Further research should consider incorporating the arm of RDT plus microscopy.

## Conclusion

RDT was more feasible than microscopy and patients with negative results received significantly more AL in the microscopy arm compared to those in the RDT arm. To realise the benefits of parasitological confirmation of malaria, service providers need to adhere to test results and they need guidance regarding management of patients with negative results.

## Competing interests

The authors declare that they have no competing interests.

## Authors' contributions

All authors conceived and designed the study; VB and FN collected, analysed, interpreted the data and drafted the manuscript; PM critically revised the manuscript. All authors read and approved the final manuscript
